# Comparison of nerve combing and percutaneous radiofrequency thermocoagulation in the treatment for idiopathic trigeminal neuralgia^☆^

**DOI:** 10.1016/j.bjorl.2015.11.006

**Published:** 2016-01-07

**Authors:** Xuanchen Zhou, Yiqing Liu, Zhiyong Yue, Deheng Luan, Hong Zhang, Jie Han

**Affiliations:** a Shandong Provincial Hospital Affiliated to Shandong University, Department of Otorhinolaryngology Head and Neck Surgery, Jinan, China; b Shandong Provincial Hospital Affiliated to Shandong University, Department of Care Gastroenterology, Jinan, China

**Keywords:** Idiopathic trigeminal neuralgia, Nerve combing, Percutaneous radiofrequency, Neuralgia idiopática do trigêmeo, Neurólise interna, Radiofrequência percutânea

## Abstract

**Introduction:**

Idiopathic trigeminal neuralgia (ITN) is a common pain disease in elderly people. Many methods have been used to alleviate the pain of patients, but few studies in the literature have compared the effect of nerve combing and percutaneous radiofrequency thermocoagulation.

**Objective:**

The purpose of this study was to describe and evaluate the clinical outcome of idiopathic trigeminal neuralgia after nerve combing (NC) and compare them with those obtained using percutaneous radiofrequency thermocoagulation (RF).

**Methods:**

The study included 105 idiopathic trigeminal neuralgia patients with similar symptom, age and underlying disease, which were divided into two groups. One group was treated by nerve combing (50 patients), the other by RF (55 cases). All patients were considered medical failures prior to the surgeries. A questionnaire was used to assess the long-term outcomes: pain relief, recurrence, complication and need for additional treatment.

**Results:**

The median duration of follow-up in both groups was 90 months. Satisfactory relief was noted in 41 patients (82%), 5 patients (10%) initially experienced pain relief, then recurred, and four patients (8%) were designated poor among the group NC. In the group RF, satisfactory relief was noted in 42 patients (76.4%). There were eight “pain free with recurrence patients (14.5%) and 5 poor cases (9.1%). No statistically significant differences existed in the outcomes between both groups (*p* > 0.05). Postoperative morbidity included dysesthesia, diplopia, partial facial nerve palsy, hearing loss, tinnitus, cerebrospinal fluid leak, meningitis and mortality.

**Conclusion:**

Nerve combing and RF are both satisfactory treatment strategies for patients with ITN. Because of the higher risk of sensory morbidity and surgical risk as open surgery, RF is preferred as the recommended procedure for patients with ITN.

## Resumo

**Introdução:**

A neuralgia idiopática do trigêmeo (NIT) é uma condição dolorosa comum em idosos. Muitos métodos têm sido usados para aliviar a dor dos pacientes, mas poucos estudos na literatura compararam o efeito de neurólise interna e termocoagulação percutânea por radiofrequência.

**Objetivo:**

O objetivo desse estudo foi descrever e avaliar o desfecho clínico de pacientes com neuralgia idiopática do trigêmeo após neurólise interna (NI) e compará-los com os obtidos usando termocoagulação percutânea por radiofrequência (RF).

**Método:**

O estudo incluiu 105 pacientes com NIT com sintomas, idade e doenças de base semelhantes, que foram divididos em dois grupos. Um grupo foi tratado por neurólise interna (50 pacientes) e o outro por RF (55 casos). Todos os pacientes haviam sido considerados fracassos terapêuticos antes das cirurgias. Um questionário foi utilizado para avaliar os resultados a longo prazo: alívio da dor, recorrência, complicações e necessidade de tratamento adicional.

**Resultados:**

A duração média do acompanhamento foi de 90 meses em ambos os grupos. Alívio satisfatório foi observado em 41 pacientes (82%); cinco pacientes (10%) experimentaram alívio inicial da dor, porém seguido de recrudescimento, e quatro pacientes (8%) apresentaram desfecho desfavorável no grupo NI. No grupo de RF, alívio satisfatório foi observado em 42 pacientes (76,4%). Houve oito pacientes livres de dor, com recorrência “LDR” (14,5%) e cinco casos com desfecho desfavorável (9,1%). Não houve diferenças significantes nos resultados entre os dois grupos (p > 0,05). Morbidade pós-operatória incluiu disestesia, diplopia, paralisia parcial do nervo facial, perda auditiva, tinnitus, fístula liquórica, meningite e óbito.

**Conclusão:**

Neurólise interna e RF são estratégias satisfatórias de tratamento para os pacientes com NIT. Em decorrência da maior morbidade sensorial e maior risco cirúrgico em uma cirurgia aberta, a RF é o procedimento mais indicado para pacientes com NIT.

## Introduction

Idiopathic trigeminal neuralgia (ITN) is the most common cephalic neuralgia afflicting the middle-aged and elderly people. It is a chronic pain syndrome characterized by paroxysmal, shock-like, stabbing, recurrent episodes of pain localized in the distribution area of one or more branches of trigeminal nerve.^1^ The most common nerve roots involved are the second and the third trigeminal nerve root. Pain usually occurs on one side of the face and is triggered by a minimal tactile stimulus like touching, wind on the face, eating or brushing, et al.^2^ Trigeminal neuralgia is more frequent in females with an incidence rate of 3–5 per 100,000.^3^, ^4^ The exact pathophysiology of TN is still in debate. Occurring from different aetiological agents, it is classified as ITN and secondary trigeminal neuralgia. ITN is caused by vascular compression of the trigeminal nerve root, which account for about 80%–90% of trigeminal neuralgia cases.^5^, ^6^ Secondary trigeminal neuralgia is caused by other factors such as tumors of posterior fossa, demyelination in multiple sclerosis and vascular disorders.

Up to now, there are no medications that completely alleviate pain caused by trigeminal neuralgia. For ITN, surgical treatments in current use include microvascular decompression (MVD), percutaneous radiofrequency thermocoagulation (RF), nerve combing (NC), percutaneous balloon compression, Gamma knife surgery, percutaneous retrogasserian glycerol rhizotomy, sensory root section of the trigeminal nerve and avulsion of peripheral branch of trigeminal nerve.^7^, ^8^, ^9^, ^10^, ^11^ The complete or reasonable pain control is 85.2%–88.9%.^9^ RF was one of the most common procedures to treat trigeminal neuralgia and has proven its worth over the past 20 years, which can initially achieve total pain relief in 80%–90% of patients.^12^ Nerve combing was a kind of surgical strategy for trigeminal neuralgia that longitudinally split the branches of trigeminal nerve using a special fiber knife according to preoperative pain locations and intraoperative findings. It has been regularly used to treat TN at our institution, having been approved by Shandong Provincial Hospital Ethic Institutional Committee.

Our previous paper has reported its reliable efficacy in patients with ITN.^11^ Here, we once more retrospectively reviewed 105 ITN patients who underwent NC or RF between 2004 and 2014 and assessed the long-term outcomes of these two procedures. The follow-up period was 48–168 months in both groups.

## Methods

### Ethics statement

Our retrospective study including 105 patients was approved by the local Ethics Institutional Committee. Each individual involved in the study signed an informed consent form authorizing the Institute to utilize their information for research purposes.

### Clinical materials

All of 105 patients had complete medical records insuring the correct diagnosis and cooperation with our follow-up postoperatively. Each patient suffered from severe, recurrent, unilateral pain and demonstrated an evident “trigger point” on the spat, upper lip or wing of nose. Although all the patients took carbamazepine as pain-controlling medication, the final pain relief was judged insufficient. They required surgical procedure for alleviating their refractory pain. A 3.0-Tesla Magnetic Resonance Imaging (MRI) scanner (General Electric Vectra, IGF Medical, Milwaukee, WI, USA) was employed to determine if there was tumor, hemangioma, multiple sclerosis or other occupying lesions compressing the root entry zone of trigeminal nerve in each patient preoperatively. In 61 cases blood vessels were found at the root of the trigeminal nerve by MRI. A total of 105 patients were divided into group NC and group RF, each group with 55 cases. Those patients with psychiatric disorders, significant cardiovascular disease, serious infectious diseases and severe endocrine diseases were been excluded. Treatment was chosen according to the patient's option. People who were unwilling to accept the increased risk of a posterior fossa craniotomy were advised to select an RF procedure. All the operations were performed by surgeons at Department of Otorhinolaryngology Head and Neck Surgery; Provincial Hospital affiliated to Shandong University, Jinan, China.

Our study data that included patient demographics, details of technical procedure, pain relief, recurrence, complications and need for additional treatment were collected by a physician who was not directly involved in the patients’ care. A questionnaire was designed for evaluation of the long-term outcome and was completed at the time of the last follow-up examination or by mail. Postoperative relief was graded according to the criteria established by University of California at San Francisco (UCSF).^13^ Pain relief was judged as “satisfactory”, “pain free, recurrence” and “poor”. “Satisfactory” assumes that a patient reported complete pain relief without the need for medication or with only using intermittent pain medication. Patients who firstly experienced complete pain relief for at least 1 month after operation, but subsequently had a recurrence of pain, were judged as “pain-free, recurrence” (PFR). Patients who reported little or no pain relief, or who experienced to persistent pain despite medication, were designated as “poor”.

### Surgical procedure

#### Nerve combing

A retrosigmoid craniotomy cerebellopontine angle exploration was performed in 50 patients under general anesthesia. Patients were placed in the lateral decubitus position with the affected side upward. A vertical skin incision about 4 cm posteriorly from the internal acoustic meatus was made, which began above the ear pinna and extended to 2 cm below the mastoid level. The soft tissues were dissected at the junction of parietal, occipital and temporal bone. A 3–4 cm retrosigmoid craniectomy with exposing of the crest of the transverse and sigmoid sinuses was performed.

The opening was shaped with superior and anterior borders constituted by the inferior margin of the transverse sinus and the posterior margin of the sigmoid sinus. The dura mater was opened with U curve and reflected against the sinuses; the cerebellar hemisphere was exposed. A gentle traction of cerebellum with spatula and draining of cerebrospinal fluid were performed, so that the cisterna was opened. Under a 10× microscope, the trigeminal nerve was identified at the posterior surface of the petrous vein below the tentorium.

The circumference of the nerve was carefully inspected to determine if the compressive vessel was present, especially at the REZ. The affected branches of trigeminal nerve, according to preoperative pain locations and intraoperative findings, were longitudinally split by a special fiber knife (manufactured by our institution). Every branch was split 2–6 fascicles from entrance to pons, depending on the size of branches. When the combing was performed under microscopic control, the special fiber knife was maintained ahead of microscope so that it would be constantly visual, avoiding possible interaction with other brain tissue. Closure was carried out by standard surgical techniques.

#### RF surgery

55 patients underwent this procedure under local or general anesthesia in the supine position. A special needle was introduced at a cutaneous puncture site 1.5 cm from the corner of mouth, through the cheek and into the foramen ovale. The trigeminal ganglion is heated to 70 for 100 s with a radiofrequency probe, producing a partial lesion.

### Statistical analysis

Values are expressed as mean ± SD and significance was evaluated by Wilcoxon Rank Sum Test, independent Student *t* test, Pearson Chi-Square, Continuity Correction or Fisher's test using commercially available software (IBM SPSS version 19.0). A *p*-value of <0.05 or <0.01 was considered as statistically significant.

## Results

A total of 105 patients underwent surgical treatments. Patients’ characteristics including sex, median age, median duration, and affected side are similar between the groups (*p* > 0.05, Table 1). The mean follow-up period was 90 months (range, 48–168 months) in both group NC and group RF.

**Table 1:** Demographic and clinical information in the 50 patients of group NC and 55 patients of group RF.

Feature	Group NC	Group RF	*p*-Value
*Total number of patients*	50	55	

*Age (years)*			
Median	48.9 ± 8.6	49.3 ± 8.7	>0.05
Range	34–72	36–73	

*Sex – M/F*	28/22	30/25	>0.05

*Age of diagnosis*	54.8 ± 10.4	56.1 ± 11.0	>0.05

*Duration of symptoms (years)*			
Median	5.6 ± 1.9	5.9 ± 1.8	>0.05
Range	1–14	1–15	

*Affected side – L/R*	23/27	26/29	>0.05

*Distribution of pain*			
V1, V2, V3	14	10	
V1, V2	8	5	
V1, V3	12	14	
V1	2	5	
V2	6	10	
V3	8	11	
Follow-up period (years)	48–168	48–168	

The statistical analysis was performed by independent Student *t* test. *p* > 0.05, not significant.

The median age of Group NC was 48.9 ± 8.6 years (range 34–72 years); 28 patients were male and 22 were female. The average age of diagnosis was 54.8 ± 10.4. Their duration of symptoms had lasted for a median of 5.8 years (range 1–14 years). 23 patients felt pain on the left side and 27 on the right side.

The median age in Group RF was 49.3 ± 8.7 years (range 36–73 years); 30 patients were male and 25 were female. The average age of diagnosis was 56.1 ± 11.0. Their duration of symptoms had lasted for a median of 6.1 years (range 1–15 years). Twenty-six patients had pain on the left side and 29 on the right side. The details of trigeminal roots affected as well as a comparison of demographic and clinical data in both groups of patients are displayed in Table 1. There was not a highly statistically significant difference among sex, median age, average age of diagnosis, median duration of the symptoms and affected side in the both groups (*p* > 0.05) (Table 1).

At the end of the follow-up, satisfactory relief were noted in 41 patients (82%), 5 patients (10%) initially experienced pain relief, then recurred, and 4 patients (8%) were judged to have a poor outcome among the group NC. Thirteen patients with recurrence had to repeat the same procedure and found satisfactory relief.

**Figure 1:**
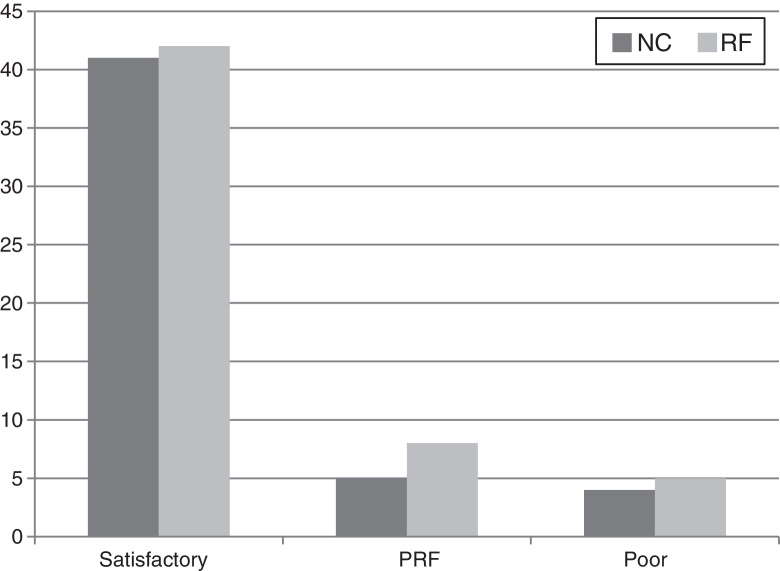
Long-term pain relief in nerve combing (NC) and percutaneous radiofrequency thermocoagulation (RF) groups (*p* > 0.05).

In the group RF, at the end of the follow-up, satisfactory relief were noted in 42 patients (76.4%). There were eight “PFR” patients (14.5%) and 5 poor cases (9.1%). Table 2 and Fig. 1 reported and compared the detailed surgical outcome in both groups, which showed there was no significant difference between group NC and RF (*p* > 0.05). Both procedures were highly effective and had similar efficacy.

**Table 2:** Surgical outcome of group NC vs. group RF (UCSF).

Pain relief outcome	Group NC (50)	Group RF (55)
Satisfactory	41 (82%)	42 (76.4%)
PFR	3 (10%)	8 (14.5%)
Poor	4 (8%)	5 (9.1%)

PFR, pain free recurrence; UCSF, University of California in San Francisco; *p* > 0.05, not significant, by Wilcoxon test.

In patients of group NC (Table 3), the postoperative morbidity included dysesthesia in 8 patients (16%), diplopia in 1 patient (2%), and partial facial nerve dysfunction in 7 patients (14%). The facial palsy improved to House–Brackmann grade I or II about 3 months after surgery. Hearing loss was recorded in only 1 patient (2%) and tinnitus in 2 patients (4%). CSF leak and meningitis were found in 3 (6%) and 4 patients (8%), respectively.

**Table 3:** Morbidity and surgical complication rates in the two patient groups.

	Group NC (50)	Group RF (55)	*p* Value
Dysethesia	8 (16%)	2 (3.6%)	0.031 (< 0.05)
Facial nerve lesion	7 (14%)	1 (1.8%)	0.048 (< 0.05)
Diplopia	1 (2%)	1 (1.8%)	1.000 (> 0.05)
Hearing loss	1 (2%)	0	0.476 (> 0.05)
Tinnitus	2 (4%)	2 (3.6%)	1.000 (> 0.05)
CSF leak	3 (6%)	0	0.209 (> 0.05)
Meningitis	4 (8%)	1 (1.8%)	0.305 (> 0.05)
Mortality	0	1 (1.8%)	1.000 (> 0.05)

CSF, cerebrospinal fluid. The statistical analysis was performed by Pearson Chi-Square, Continuity Correction or Fisher's exact test. *p* > 0.05, not significant

In patients of group RF, the most frequent complications were dysesthesia and tinnitus, which developed in 2 patients (3.6%). Diplopia, meningitis and mortality were all recorded in only 1 patient (1.8%). This patient suffered from coma because of older age (69 yrs), hypertension and diabetes, and died ultimately of pulmonary infection. This patient was classified into “poor”.

The statistical analysis showed that no significant differences existed between the patients in the 2 groups regarding the surgery-related complications (*p* > 0.05), except for dysesthesia and facial nerve palsy (*p* < 0.05).

## Discussion

TN is the most common form of facial pain in people older than 50 years of age. Its classical symptoms are paroxysmal shock-like, stabbing, recurrent episodes of pain in the distribution of one or more divisions of trigeminal nerve.^1^ For ITN, the vascular compression hypothesis is now generally accepted, both because of the intraoperative findings and the better long-term results of decompression surgery. Superior cerebellar artery compression is seen most frequently. Medical treatment still remains the first line of approach. However, nearly half of patients suffering from ITN eventually required surgical methods for pain relief.^14^

Li et al. in 1995 first used nerve combing to treat TN patients without vascular compression. Ma et al. described ten patients without vascular compression who underwent NC and achieved satisfactory long-term relief in 70%.^15^ Few reports have reported on treatment of TN patients with vascular compression by trigeminal NC. Our recent publication showed this procedure could result in 87.5% satisfactory pain relief in 32 TN patients with vessel compression. In this report, we obtained 82% satisfactory pain relief rate.

RF that was first performed by Kirchner in 1931, then modified by Sweet in 1965, has been also considered as a safe and effective treatment for ITN.^16^ As a least invasive option, its advantages include shorter procedure duration and the need for only local anesthesia. But the relief rates were different according to different reported studies. Liu Chao documented in his review that RF can achieve total pain relief in 96% of patients after 6 months to 2 years’ follow-up.^17^ Bidkar Prasanna showed 84.6% of 39 patients in the RF group experienced excellent pain relief, while the recurrent rate was as high as 51.5% at the end of the study period.^18^ Fraioli achieved a higher success rate (99.2%) and 10% recurrence rate with RF.^19^ In our experience, 76.4% of patients had satisfactory pain control at the last follow-up examination. This compares differently with previously published results. The reason may be related to single and fixed temperature and time (70 s for 100 s) used on every patient. Other authors employed temperatures and time ranging from 60 to 80 s and 60 to 90 s.^12^, ^19^, ^20^ However, we observed a significantly lower rate of pain recurrence (14.5%) in our series than the rates reported in other reports, which might be associated with the surgeon's degree of proficiency.

The morbidity rate and the surgery-related complication rate in the current series seemed to be similar in both group NC and RF (*p* > 0.05), except for dysesthesia and facial nerve palsy (*p* < 0.05). The former may be associated with longer procedure duration in the nerve combing surgery and the latter may relate to damaging of the facial nerve in the open surgery. Dysesthesia was a common side effect after the RF surgery. Erdine et al. reported a prospective, randomized study about RF and described that all of the 20 patients suffered from mild hypoesthesia and paresthesia after the procedure.^21^

This series involved fewer patients than in our study, but dysesthesia was still the most frequent complication among all the side effects in both groups. It is difficult to avoid injuring the normal trigeminal function from heat. Hearing loss developed in 1 patient of group NC. The reason may be damaging the cochlear nerves, which also lead to tinnitus. One mortality was recorded in the group RF, because of concomitant serious conditions, such as diabetes and hypertension, and not the surgery itself. No doubt that NC as an open procedure has led to more CSF and meningitis morbidity than RF, but there was not significant difference observed (*p* > 0.05).

Our study also suffers from some limitations. First, the retrospective nature of our study makes the study subject to a recall bias and is limited to available data in the patient charts. Second, the treatment allocation to groups was not random, but rather based on clinician and patients’ preference. Despite these limitations, we believe that our results are valid and will help physicians involved in the care of these difficult-to-treat patients. A prospective randomized study designed to compare NC and RF in patients with ITN would certainly be of value to validate and strengthen these results.

## Conclusion

Nerve combing and RF are both satisfactory treatment strategies for patients with ITN. From our analysis above, both NC and RF have similar pain relief rates, recurrent rates and complications. But NC carries higher sensory and facial palsy morbidity than RF (*p* < 0.05). In addition, NC is an open retrogasserian surgery with much higher surgical risk, compared to minimally invasive RF. Some elderly patients may be not suitable for this type of surgical intervention. Therefore, RF is preferable to NC in patients with ITN.

## Conflicts of interest

The authors declare no conflicts of interest.
